# Weight Change in Post-Menopausal Women with Breast Cancer during Chemotherapy—Perspectives on Nutrition, Activity and Bone Metabolism: An Interim Analysis of a 5-Year Prospective Cohort

**DOI:** 10.3390/nu13082902

**Published:** 2021-08-23

**Authors:** Kristian Buch-Larsen, Trine Lund-Jacobsen, Michael Andersson, Peter Schwarz

**Affiliations:** 1 Department of Endocrinology and Metabolism, Rigshospitalet, 2100 Copenhagen, Denmark; trine.lund-jacobsen@regionh.dk (T.L.-J.); peter.schwarz@regionh.dk (P.S.); 2 Department of Oncology, Rigshospitalet, 2100 Copenhagen, Denmark; michael.andersson@regionh.dk; 3 Faculty of Health Sciences, University of Copenhagen, 2100 Copenhagen, Denmark

**Keywords:** breast cancer, metabolism, body weight, chemotherapy, nutrition, exercise, patient-reported outcomes

## Abstract

Women with breast cancer are a growing population due to improved screening and treatment. It has been described that chemotherapy can negatively affect patients’ metabolism. The aim of this study is to assess weight gain during chemotherapy treatment in an interim analysis on an ongoing prospective cohort of women with early breast cancer. To help untangle the many possible reasons for weight change, we examine blood tests, Patient-Reported Outcomes (PROs), and bone mineral density (BMD). We find that the 38 women that have measurements taken after chemotherapy have an average weight gain of 1.2 kg although not significant. Together with this, there is a significant drop in HDL cholesterol, an increase in triglycerides, and a non-significant tendency towards decreased insulin sensitivity. PROs show that although the women experience more pain and fatigue, they have higher activity levels. BMD is at an expected level according to age. All in all, we see an increased focus on physical activity and nutrition, leading to less severe metabolic changes as previously reported. However, even though more measures are taken, we still see an overall negative metabolic impact with unknown long-term implications.

## 1. Introduction

The treatment of breast cancer (BC) has steadily improved over the last decades and even though we see more cases of breast cancer each year, survival rates are increasing in Europe, mainly due to screening programs and improvements in treatment [1]. Today, we see a global 5-year survival rate of 73% for all stages of the disease, with higher numbers in developed countries and for lower stages [2]. Since BC accounts for approximately 25% of all new cancers in women [3], that amounts to a considerable amount of long-term breast cancer survivors. Considering this positive development, it is imperative that the long-term consequences of the oncological treatment are further investigated. This can, in turn, provide clinicians with a better understanding of who to observe closer and help counteract possible negative effects of treatment.

It has been described for close to half a century that women receiving adjuvant chemotherapy are compromised metabolically [4,5,6]. This is mainly observed as an average weight gain of 3–5 kg, but with a great variance among individual patients [4,5,6]. The specific mechanism for this negative change has not been fully elucidated, but many theories exist [7]. Concurrently, most post-menopausal women with hormone receptor positive early BC will receive aromatase inhibitors, which are known to further deteriorate the metabolic profile, specifically the lipid profile [8].

Diet and exercise have been an area of increasing interest when looking at patients with BC [9]. Thus, when examining metabolism it is important to look at these aspects and for this Patient-Reported Outcomes (PROs) is often the method of choice [10]. Pain and fatigue are subjective phenomena, which is why the assessment must be obtained directly from the individual through patient-reported questionnaires. Patient-reported results are useful and have been shown to have prognostic value [11,12]. Cancer-related fatigue, as well as pain, are multidimensional concepts affecting the physical (less energy and more need for sleep), cognitive (decreased concentration and attention), and affective (decreased motivation) domains. Cancer-related fatigue and pain limit the health-related quality of life of BC survivors and their reincorporation into normal life, including their ability to return to work [13,14].

When studying metabolic changes during BC treatment, the overall endocrinological status of these patients might be compromised. It is well described that aromatase inhibitors (AI), given to post-menopausal women with estrogen receptor-positive breast cancer, might cause a loss of bone mineral density (BMD) [15,16,17,18]. It is therefore interesting in the perspective of overall endocrine status to examine bone status before start of AI.

In order to investigate this metabolic derangement, we have set up a prospective cohort of post-menopausal women, all receiving chemotherapy, and follow them prospectively with an endocrine perspective. In this study, we present the first results from this cohort, focusing on metabolic changes during chemotherapy as well as a questionnaire-based status on these women with regards to diet, physical exercise, and general well-being as possible explanations for metabolic changes.

The aim of this study was to assess weight gain during chemotherapy and relate this to changes in biochemistry and a cross-sectional description of patient-reported physical activity, dietary intake, and bone quality.

## 2. Materials and Methods

**Figure 1:**
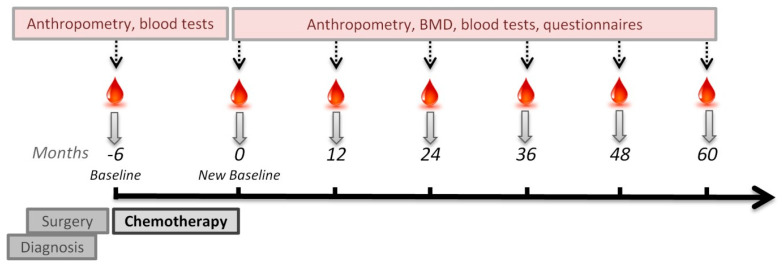
Study design. BMD: Bone Mineral Density.

Patients followed at the Department of Oncology, Rigshospitalet were asked to participate in a 5-year prospective cohort if they were between 50 and 70 years of age, post-menopausal, and were scheduled to receive chemotherapy; see Figure 1 for details on study setup. Exclusion criteria were known endocrine disease (e.g., diabetes mellitus, osteoporosis, or thyroid disease) as well as cancer treatment prior to the current diagnosis of BC and disseminated BC.

In this interim analysis we have data from −6 months (baseline) and 0 months (new baseline).

If patients agreed to participate, they got blood drawn for a broad array of metabolic tests including, but not limited to, glucose metabolism, lipids, and calcium homeostasis. After the end of chemotherapy patients were examined at new baseline with DXA scan, repeated blood tests, questionnaires, and nerve conducting tests. This will be performed yearly for five years post-chemotherapy. This is an early report on preliminary data from this ongoing cohort. It has been approved by the local ethical committee and is registered at clinicaltrials.gov.

Height and weight measurements were performed before start of chemotherapy at the Department of Oncology and again at the time of DXA scan. BMD measurements using DXA were performed after chemotherapy. BMD was measured at lumbar spine (mean of L1-L4), femoral neck, and total hip. DXA accurately determines 2-dimensional BMD (g/cm^2^) and is used to detect an increased risk of incurring an osteoporotic fracture [19]. A Hologic DiscoveryTM QDR Series scanner was used, and the same laboratory technician performed all analyses. Daily phantom measurements were conducted and calibration according to standard procedure. According to the manufacturer, the coefficient of variation of the total BMD is approximately 1% (Europe H. Hologic Osteoporosis Assessment. Reference Manual. 2006; Document No. Man-00214).

All blood samples were obtained from venepuncture fasting before 10:00 AM in the antecubital vein and processed and analyzed shortly after, at the central laboratory at Rigshospitalet, Denmark. Plasma was analyzed before and after patients received chemotherapy and included plasma (p)-25-hydroxyvitamin D (p-25OHD), p-creatinine, p-alkaline phosphatase, p-albumin. p-ionized calcium (p-Ca^2+^) and p-parathyroid hormone (p-PTH).

The questionnaires used are Food Frequency Questionnaire (FFQ) [20], the European Organization for Research and Treatment of Cancer (EORTC) Quality of Life (QLQ-C30)(EORTC QLQ-C30) [21], and the Medical Outcomes Study 36-item Short-Form Health Survey (SF-36) [22].

General Health-Related Quality of Life (HRQoL) was assessed using the following two questionnaires:

The EORTC QLQ-C30 questionnaire is a cancer-specific, multi-dimension, self-administrated questionnaire designed for use in clinical trials [21] that contains 30 questions. The EORTC QLQ-C30 core questionnaire contains a global health scale, five functional scales (physical, role, emotional, cognitive, and social), three symptom scales (fatigue, nausea/vomiting, and pain), and six single items (dyspnea, insomnia, appetite loss, constipation, diarrhea, and financial difficulties). For functional scales, scores computed range from 0 to 100, with higher values representing a higher level of problems [21].

The SF-36 Health Survey questionnaire is a generic, multi-dimensional, self-administrated questionnaire [22] that measures two major health concepts (physical and mental health) with 36 questions. The SF-36 contains eight multi-item scales: physical functioning, role-physical, role-emotional, bodily pain, social functioning, mental health, vitality, and general health perceptions. Each scale is scored from 0–100, with higher scores representing a more favorable level of health [22].

Data on dietary intake were assessed using the 48-item FFQ which had also been used in a previous cross-sectional epidemiological survey [20]. Participants were asked to recall their usual frequency of dairy intake at the new baseline in the study. It included questions about the type of bread, spread, and fats used for cooking. The participants were further asked how often 27 food items (including hot meals, accompaniment to hot meals, vegetables, etc.) were consumed 24 h dietary recall choosing between four possible responses: 0 days/week, 1–2 day/week, 3–4 day/week, or 5–7 day/week. For fruit intake, eight possible responses were ranging from none to more than six pieces a day [20].

Statistically, we based the power calculation on being able to detect a 4 kg change in weight, for this we would need 25 participants with measurements before and after chemotherapy. Statistical analysis was performed in R and we performed paired t-tests regarding biochemistry.

## 3. Results

### 3.1. Baseline Characteristics

All patients were diagnosed with breast cancer, and all received chemotherapy. The baseline characteristics of disease and treatment are shown in Table 1. Most patients presented with an invasive ductal carcinoma (86.8%) and tumor stage 2 (52.6%) or 3 (38.8%). The majority were treated with lumpectomy (63.2%) and all received paclitaxel and most received cyclophosphamide and epirubicine prior (86.8%).

### 3.2. Anthropometry

Anthropometric and biochemistry results are shown in Table 2. Average age at inclusion was 58.9 years, suggesting an even distribution within inclusion range. We see an average increase in body weight of 1.2 kg, this is not significant with a *p*-value of 0.29. We also see a (−1.5 cm) significant change in height. We did not find any vertebral crush fractures in any of the patients, so most likely due to the height being measured at two different sites.

### 3.3. Metabolism

The metabolic markers show significant changes with regard to p-HDL which drops 0.16 mmol/L (*p* < 0.01) and p-triglycerides that increase by 0.20 mmol/L (*p* = 0.01) during chemotherapy. P-LDL and p-total cholesterol remain unchanged. Glucose metabolism is generally unchanged when considering p-glucose and HbA1c where no change is observed. Insulin is increased by 17.4 pmol/L (*p* = 0.10) but remains non-significant.

### 3.4. General Biochemistry

As expected, we see significant decreases in B-hemoglobin of 0.3 mmol/L (*p* < 0.01) and B-leukocytes of 1.2 10^9^/L (*p* < 0.01). We also see kidney function being slightly decreased with a small but significant increase in P-Creatinine of 3.6 µmol/L (*p* < 0.01)

### 3.5. Questionnaires

In this study, we only present the results from the first visit after chemotherapy. Therefore, this is a cross-sectional representation of the habits and Health-Related Quality of Life measurements of these women.

The Health-Related Quality of Life measurements in SF-36 (see Table 3) shows that the women (n = 33) reported high physical activity (mean 81.06, SD 16.04), high fatigue (mean 59.24, SD 20.97), high bodily pain (mean 80.98, SD 18.72), and high nausea/vomiting (mean 79.76, SD 13.36). It is similar to the reported outcomes in the EORTC QLQ-C30 questionnaire (see Appendix A).

Two-thirds (85%) of the women (n = 33) reported in the FFQ (see Table 4 for highlighted results and Appendix B for full details) that they eat 3 or 4 meals pr. day. Furthermore, two-thirds (72%) reported that they do not eat any kind of white bread, but they do not even eat light or dark rye bread or whole meal bread. A little bit more than half (58%) of the women reported that they do not eat any kind of fats on their bread. More than half (51–64%) reported that they eat beef/veal, pig, poultry, or egg dishes 1–2 times a week. Nearly all (97%) reported that they do not use solids for cooking. Half (46–58%) reported that they often have eaten potatoes, pasta, rice, or bulgur for their hot meals in the last week. Nearly all (91%) reported that they usually eat 1 piece of fruit per day.

### 3.6. Calcium Metabolism and Bone Mineral Density

We find clinically insignificant decreases in p-ionized calcium of 0.02 mmol/L (*p* < 0.01) and corresponding increase in p-PTH of 0.97 pmol/L (*p* = 0.03) (see Table 2). This is in line with some patients having started anti-resorptive treatment prior to new baseline visit, but no indications of bone metastases. When we examine BMD in the patients after chemotherapy (Table 5), we find mean BMD at the spine of 0.923 g/cm^2^, total hip 0.836 g/cm^2^, and femoral neck 0.717 g/cm^2^.

Examining the T-scores of the spine we find that 17 have osteopenic values and 4 have osteoporotic values, with the remaining 17 having normal T-scores and a mean T-score of −1.15. Compared to age-matched controls there is no difference, as seen on the Z-scores.

## 4. Discussion

Our study does not show a significant increase in body weight, but on average our patients put on 1.2 kg during chemotherapy treatment. This is in agreement with the vast majority of the literature, which shows that during the chemotherapy treatment for BC, patients gain weight [4,5]. The weight gain that we find does not, however, match the amount usually reported as between 3–5 kg.

We also show a negative impact on HDL and triglycerides as well as a non-significant increase in insulin resistance. That BC treatment can worsen glucose metabolism is well known [7]. It is also worth keeping in mind that most (70–80%) post-menopausal women with BC receive AI treatment due to being estrogen receptor-positive [23]. This treatment has a well-known negative effect on lipids [8]. There is potential of a compounded long-term negative effect on lipids [24]. Women during midlife gain approximately 0.5 kg per year irrespective of initial weight [25,26]. This is important when evaluating the long-term weight gains, especially during AI treatment. Compared to the observed weight gain over a 4-to-6-month period in this study, we still see a larger increase than what can normally be accounted for by normal age-related weight gain.

The questionnaire results we report in this paper illuminate some possible explanations for why our findings we not as pronounced as expected. It has been widely reported that pain (with time potentially chronic pain) is the most frequent patient-reported consequence of BC treatment [27,28,29]. In our study, we find that even then the BC women reported a higher level of bodily pain than the general population they also reported a higher Physical Activity level than the general population, providing a possible explanation for the less pronounced weight gain.

This increase in physical activity may be a consequence of an increased focus on the benefits of physical activity by treating physicians. This in turn is due to the many studies that for decades have demonstrated the significance of physical activity in women with BC [26]. Several studies have shown that there is a link between physical activity and increased health-related quality of life in long-term BC survivors [30,31,32]. Furthermore, physical activity has been shown in previous research to help retain bone density and musculoskeletal health [33,34].

Regarding dietary intake, breast cancer patients reported that they have a relatively low-fat diet with regular consumption of fiber such as vegetables and high-quality protein intake. This kind of diet is reported in other studies as beneficial and high consumption of saturated fats could be associated with a higher risk of mortality [35]. A systematic review from 2020 shows that the low-fat diet and healthy quality diet should be recommended but none of the food categories (meat, dairy products) should be eliminated in cancer patients’ diet [35]. The BC patients in our study also reported that they eat meat every week, but not daily.

BMD results are normal for age, which supports earlier work done by Christensen et al. [36]. Due to age, we still see 21 out of 38 with osteopenic (or worse) BMD scores at the spine. Since this is before potential start with AI treatment, it is important to monitor patients who do not receive standard treatment with bisphosphonates since AI is known to decrease BMD otherwise [18].

The main limitation of this study is in the number of participants due to its interim nature. In addition, questionnaires are often difficult to assess, but as noted previously are often the only way to quantify certain aspects of patient health.

In the long run, we hope to improve our knowledge of the metabolic consequences of BC treatment, not only chemotherapy but also potential AI treatment and how it affects women. There are also other avenues to explore, such as changes in thyroid hormone levels. Some studies have shown that oncological treatment can affect TSH (and peripheral hormones), and possibly increase autoimmunity [37,38]. This is interesting to look further into and could potentially explain several of the changes. As we see the patients at further time points, we hope to enhance knowledge on post-diagnostic diet in patients with BC, and whether it changes over the years.

## 5. Conclusions

In conclusion, it is worth noting that even though we see increases in physical activity, we still see non-significant increases in weight and decreased insulin sensitivity while still finding a significant drop in HDL and increased triglycerides. So, we still observe a metabolic deterioration and further studies are needed to provide knowledge on how best to avoid negative metabolic effects in the long run.

## Figures and Tables

**Table 1:** Disease and treatment characteristics. ER: Estrogen Receptor; HER2: Human Epidermal Growth Factor Receptor 2.

Disease Characteristics	N = 38
**Histology**	
Ductal invasive	33 (86.8%)
Lobular invasive	4 (10.5%)
Other	1 (2.6%)
**Tumor stage**	
1	1 (2.6%)
2	20 (52.6%)
3	14 (38.8%)
Unknown	3 (7.9%)
**Lymph node status**	
0	14 (36.8%)
1–3	12 (31.6%)
4+	4 (10.5%)
Unknown	8 (21.1%)
**ER-receptor status**	
Positive	31 (81.6%)
Negative	7 (18.4%)
**HER2-receptor status**	
Yes	12 (31.6%)
No	26 (68.4%)
**Treatment Characteristics**	
**Surgery**	
Mastectomy	14 (36.8%)
Lumpectomy	24 (63.2%)
**Cyclophosphamide + Epirubicine**	
Yes	33 (86.8%)
No	5 (13.2%)
**Paclitaxel**	
Yes	38 (100.0%)
No	0 (0.0%)
**Radiation treatment**	
Yes	30 (78.9%)
No	8 (21.1%)

**Table 2:** Anthropometry and biochemistry.

(N = 38)	Baseline (mean ± SD)	New Baseline (mean ± SD)	*p*-Value
Age (years)	58.9 ± 5.0	N/A	N/A
Weight ^1^ (kg)	75.7 ± 13.6	76.9 ± 13.5	0.29
Height ^1^ (cm)	168.4 ± 5.9	166.9 ± 5.9	<0.01
B-Hemoglobin (mmol/L)	8.3 ± 0.6	8.0 ± 0.5	<0.01 *
B-Leukocytes (10^9^/L)	5.8 ± 1.1	4.6 ± 1.1	<0.01 *
P-Albumin (g/L)	39.7 ± 2.5	38.6 ± 2.2	<0.01 *
P-Creatinine (µmol/L)	66.3 ± 9.9	69.9 ± 11.4	<0.01 *
P-Ionized Calcium ^2^ (mmol/L)	1.26 ± 0.05	1.24 ± 0.05	<0.01 *
P-Phosphate (mmol/L)	1.14 ± 0.17	1.15 ± 0.16	0.69
P-Magnesium (mmol/L)	0.89 ± 0.05	0.89 ± 0.06	0.74
P-Alkaline Phospatase (U/L)	72.4 ± 19.7	74.9 ± 33.4	0.47
P-PTH ^2^ (pmol/L)	5.24 ± 2.30	6.21 ± 2.41	0.03 *
P-25-OH-vitamin D (nmol/L)	59.6 ± 23.0	73.3 ± 24.8	<0.01 *
P-HDL (mmol/L)	1.81 ± 0.49	1.65 ± 0.40	<0.01 *
P-LDL (mmol/L)	3.72 ± 1.05	3.73 ± 0.96	0.81
P-Total cholesterol (mmol/L)	5.71 ± 1.11	5.73 ± 1.06	0.88
P-Triglycerides (mmol/L)	1.24 ± 0.57	1.44 ± 0.63	0.01 *
P-glucose (mmol/L)	5.5 ± 0.5	5.5 ± 0.6	0.65
HbA1c (mmol/mol)	35.5 ± 2.9	35.6 ± 3.7	0.68
P-Insulin (pmol/L)	69.9 ± 43.4	87.3 ± 32.8	0.10

^1^ Weight and height measurements performed at Department of Oncology at baseline, all subsequent measurements performed at the Department of Endocrinology. ^2^ Some patients had started zoledronic acid prior to new baseline visit. * Marks significance below 0.05.

**Table 3:** SF 36 survey results and reference population.

SF-36	New Baseline (N = 33) Mean ± SD	Reference * (N = 313) Mean ± SD
**Physical functioning**	81.1 ± 16.0	75.4 ± 23.2
**Role physical**	50.0 ± 41.7	62.4 ± 42.4
**Bodily pain**	81.0 ± 18.7	65.1 ± 26.1
**General Health**	68.5 ± 12.1	67.3 ± 23.6
**Social functioning**	77.7 ± 24.4	84.9 ± 21.9
**Role emotional**	70.7 ± 38.3	77.5 ± 35.4
**Energy/fatigue (physical summary)**	59.2 ± 21.0	43.9 ± 11.9
**Emotional well-being (mental summary)**	79.8 ± 13.4	53.1 ± 9.4

* Garratt AM and Stavem K. “Measurement properties and normative data for the Norwegian SF-36: results from a general population survey” (2017) Health and Quality of Life Outcomes.

**Table 4:** Food Frequency Questionnaire survey results—selected results. See Appendix B for full details.

Food Frequency Questionnaire (Selected Results)	No. of Patients (%)
**How many meals do you eat a day? (Fruit, cake, bread, etc. are perceived as meals, while liquids and sweets are not considered as meals)**	
1 meal	0 (0%)
2 meals	0 (0%)
3 meals	15 (46%)
4 meals	13 (39%)
5 meals	4 (12%)
6 or more meals	1 (3%)
**What kind of bread do you eat most often?**	
**White bread**	
Yes	6 (18%)
No	24 (72%)
**What kind of fats do you use on the bread?**	
**None**	
Yes	19 (58%)
No	14 (42%)
**How often have you eaten the following hot meals in the last week?**	
**Poultry**	
0 times	7 (21%)
1–2 times	20 (61%)
3–4 times	6 (18%)
5–7 times	0 (0%)
n/a	0 (0%)
**Vegetable/vegetarian dishes**	
0 times	9 (27%)
1–2 times	13 (40%)
3–4 times	5 (15%)
5–7 times	6 (18%)
n/a	0 (0%)
**What solids (e.g., butter, lard, margarine) do you use for cooking?**	
**None**	
Yes	1 (3%)
No	32 (97%)
**How often have you eaten potatoes/pasta/rice etc. for your hot meals in the last week?**	
**Pasta**	
0 times	11 (33%)
1–2 times	15 (46%)
3–4 times	4 (12%)
5–7 times	0 (0%)
n/a	3 (9%)
**How much fruit do you usually eat per day/week? (1 serving = 1 piece or 1 dl)?**	
None	3 (9%)
1–2 a week	7 (21%)
3–4 a week	3 (9%)
5–6 a week	6 (18%)
1–3 a day	9 (27%)
3–4 a day	5 (16%)
5–6 a day	0 (0%)
More than 6 a day	0 (0%)

**Table 5:** DXA results at new baseline visit. BMD (Bone Mineral Density); TBS (Trabecular Bone Score).

N = 38	New Baseline		
	BMD (g/cm^2^) Mean ± SD	T-Score Mean ± SD	Z-Score Mean ± SD
BMD spine	0.923 ± 0.118	−1.15 ± 1.08	0.27 ± 1.01
BMD left hip total	0.836 ± 0.102	−0.97 ± 0.94	0.03 ± 0.85
BMD left femoral neck	0.717 ± 0.101	−1.21 ± 0.91	0.07 ± 0.87
TBS	1.354 ± 0.086	N/A	N/A

## Data Availability

Data in the project is still being collected, but all data used in the study is available by contacting the authors.

## Appendix A

**EORTC QLQ-C30 Domain** **N = 33**	**BCP Age 50–70 Female** **Mean ± SD**
Function subscales	
Physical function	87.7 ± 14.9
Role function	79.3 ± 22.9
Emotional function	81.1 ± 18.6
Cognitive function	78.3 ± 21.9
Social function	83.8 ± 20.3
Symptom subscales/items	
Fatigue	28.6 ± 21.7
Nausea/vomiting	3.5 ± 6.8
Pain	15.7 ± 19.7
Dyspnoea	14.1 ± 21.8
Insomnia	30.3 ± 31.1
Appetite loss	6.1 ± 17.3
Constipation	4.0 ± 10.9
Diarrhoea	15.2 ± 24.7
Financial difficulties	10.1 ± 23.9
Global health/Quality of Life	70.2 ± 14.8

## Appendix B

**Food Frequency Questionnaire**	**No. of Patients (%)**
How many meals do you eat a day? (Fruit, cake, bread, etc. are perceived as meals, while liquids and sweets are not considered as meals)	
1 meal	0 (0%)
2 meals	0 (0%)
3 meals	15 (46%)
4 meals	13 (39%)
5 meals	4 (12%)
6 or more meals	1 (3%)
What kind of bread do you eat most often?	
Light or dark rye bread	
Yes	18 (55%)
No	15 (45%)
Wholemeal bread	
Yes	20 (61%)
No	13 (39%)
White bread	
Yes	6 (18%)
No	24 (72%)
White bread coarse	
Yes	2 (6%)
No	31 (94%)
White bread Italian	
Yes	1 (3%)
No	32 (97%)
What kind of fats do you use on the bread?	
None	
Yes	19 (58%)
No	14 (42%)
Minarine	
Yes	0 (0%)
No	33 (100%)
Vegetable margarine	
Yes	1 (3%)
No	32 (97%)
Butter	
Yes	11 (33%)
No	22 (67%)
Mixed Spreadable	
Yes	10 (30%)
No	23 (70%)
Lard	
Yes	1 (3%)
No	32 (97%)
How often have you eaten the following foods with bread in the last week?	
Cheese 0–7% fat	
0 times	18 (55%)
1–2 times	1 (3%)
3–4 times	2 (6%)
5–7 times	1 (3%)
n/a	11(33%)
Cheese 27–38% fat	
0 times	6 (19%)
1–2 times	8 (24%)
3–4 times	9 (27%)
5–7 times	8 (24%)
n/a	2 (6%)
Meat	
0 times	5 (15%)
1–2 times	13 (40%)
3–4 times	7 (21%)
5–7 times	5 (15%)
n/a	3 (9%)
Fish	
0 times	7 (21%)
1–2 times	14 (43%)
3–4 times	7 (21%)
5–7 times	2 (6%)
n/a	3 (9%)
Egg	
0 times	4 (12%)
1–2 times	17 (51%)
3–4 times	6 (19%)
5–7 times	4 (12%)
n/a	2 (6%)
Mayonnaise salads	
0 times	20 (61%)
1–2 times	8 (24%)
3–4 times	0 (0%)
5–7 times	0 (0%)
n/a	5 (15%)
Vegetables	
0 times	3 (9%)
1–2 times	7 (21%)
3–4 times	8 (24%)
5–7 times	13 (40%)
n/a	2 (6%)
Marmalade/honey	
0 times	12 (37%)
1–2 times	9 (27%)
3–4 times	5 (15%)
5–7 times	4 (12%)
n/a	3 (9%)
How often have you eaten the following hot meals in the last week?	
Beef/veal	
0 times	8 (24%)
1–2 times	21 (64%)
3–4 times	3 (9%)
5–7 times	0 (0%)
n/a	1 (3%)
Pig	
0 times	16 (49%)
1–2 times	17 (51%)
3–4 times	0 (0%)
5–7 times	0 (0%)
n/a	0 (0%)
Poultry	
0 times	7 (21%)
1–2 times	20 (61%)
3–4 times	6 (18%)
5–7 times	0 (0%)
n/a	0 (0%)
Mincemeat	
0 times	29 (88%)
1–2 times	0 (0%)
3–4 times	0 (0%)
5–7 times	0 (0%)
n/a	4 (12%)
Egg Dishes	
0 times	13 (40%)
1–2 times	17 (51%)
3–4 times	0 (0%)
5–7 times	1 (3%)
n/a	2 (6%)
Vegetable/vegetarian dishes	
0 times	9 (27%)
1–2 times	13 (40%)
3–4 times	5 (15%)
5–7 times	6 (18%)
n/a	0 (0%)
Porridge	
0 times	25 (76%)
1–2 times	2 (6%)
3–4 times	1 (3%)
5–7 times	2 (6%)
n/a	3 (9%)
Ready meals (e.g., pizza, burger and other non-home cooked meals)	
0 times	23 (70%)
1–2 times	6 (18%)
3–4 times	1 (3%)
5–7 time	0 (0%)
n/a	3 (9%)
Sausages	
0 times	25 (76%)
1–2 times	4 (12%)
3–4 times	0 (0%)
5–7 times	0 (0%)
n/a	4 (12%)
What solids do you use for cooking?	
None	
Yes	1 (3%)
No	32 (97%)
Store	
Yes	2 (6%)
No	31 (94%)
Vegetable magazine	
Yes	4 (12%)
No	29 (88%)
Butter	
Yes	14 (42%)
No	19 (58%)
Among spreadable	
Yes	4 (12%)
No	29 (88%)
Fat	
Yes	0 (0%)
No	33 (100%)
Cooking/salad oil (rapeseed oil)	
Yes	11 (33%)
No	22 (67%)
Olive oil	
Yes	24 (73%)
No	9 (27%)
Corn-/sunflower-/window kernel oil	
Yes	7 (21%)
No	26 (79%)
Other things	
Yes	2 (6%)
No	31 (94%)
How often have you eaten potatoes/pasta/rice etc. for your hot meals in the last week?	
Potatoes	
0 times	7 (21%)
1–2 times	16 (49%)
3–4 times	8 (24%)
5–7 times	0 (0%)
n/a	2 (6%)
Pasta	
0 times	11 (33%)
1–2 times	15 (46%)
3–4 times	4 (12%)
5–7 times	0 (0%)
n/a	3 (9%)
Rice/bulgur etc.	
0 times	8 (24%)
1–2 times	19 (58%)
3–4 times	2 (6%)
5–7 times	1 (3%)
n/a	3 (9%)
Bread	
0 times	9 (27%)
1–2 times	14 (42%)
3–4 times	6 (18%)
5–7 times	0 (0%)
n/a	4 (12%)
Other things	
0 times	13 (39%)
1–2 times	4 (12%)
3–4 times	1 (3%)
5–7 times	3 (9%)
n/a	12 (37%)
How often have you eaten vegetables in addition to your hot meals in the last week?	
Salad or raw vegetables	
0 times	3 (9%)
1–2 times	11 (33%)
3–4 times	10 (31%)
5–7 times	9 (27%)
n/a	0 (0%)
Cooked vegetables	
0 times	4 (12%)
1–2 times	14 (42%)
3–4 times	4 (12%)
5–7 times	6 (18%)
n/a	5 (16%)
Vegetables in hot dishes	
0 times	5 (16%)
1–2 times	8 (24%)
3–4 times	9 (27%)
5–7 times	9 (27%)
n/a	2 (6%)
Other things	
0 times	11 (36%)
1–2 times	2 (6%)
3–4 times	1 (1%)
5–7 times	1 (1%)
n/a	18 (56%)
How much fruit/fruit juicedo you eat/drink per day/week? (1 serving = 1 piece or 1 dl)?	
None	3 (9%)
1–2 a week	7 (21%)
3–4 a week	3 (9%)
5–6 a week	6 (18%)
1–3 a day	9 (27%)
3–4 a day	5 (16%)
5–6 a day	0 (0%)
More than 6 a day	0 (0%)
